# Death from Chloroform in Boston

**Published:** 1874-01

**Authors:** 


"Death from Chloroform in Boston.—The jury presented the following verdict:

"That Mary F. Crie came to her death on Monday, the 10th day of November, 1873, between eleven A. M. and one P. M., in the office of Dr. Charles Eastham, a dentist, No. 25 Tremont street, Boston, and that her death was caused by the inhalation of chloroform administered in a mixture of chloroform and ether by the said Dr. Eastham. The jury use this opportunity to caution the public against the inhalation of so dangerous an agent as chloroform for the production of insensibility to pain. In the opinion of the jury the inhalation of sulphuric ether is safe, while the inhalation of chloroform, either alone or mixed, is always attended with danger."

This case has attracted much attention, not only from the attempt made just after the accident to pass the death off as one from ether, but also, when it became evident that it was due to chloroform, from anxiety to see what would be the conclusions of a Boston jury. The verdict is all that could be desired, as it expresses emphatically the feeling of the profession, and we do not find fault that Dr. Eastham was spared the well-deserved censure which he must have expected. The misfortunes of the past should be remembered only as warnings for the future. The use of chloroform is least justifiable where ether is best known; there is less excuse for its use in America than in Europe, and least of all in this city. After this verdict nothing but very exceptional circumstances will warrant its administration. It appears in the evidence that several dentists are in the habit of giving which ever anæsthetic they see fit, regardless of the request of the patient. We hope that this custom is not general, and would advise any who may persist in it not to be too sure that after another patient, who shall have asked for ether, has been killed by chloroform, the verdict may not contain, besides other disagreeable words, the adjective "criminal."

